# Prediction of muscular architecture of the rectus femoris and vastus lateralis from EMG during isometric contractions in soccer players

**DOI:** 10.1186/2193-1801-2-548

**Published:** 2013-10-18

**Authors:** Bhawesh Chauhan, Maher A Hamzeh, Antonio I Cuesta-Vargas

**Affiliations:** Department of Life Sciences, Centre for Sport and Exercise Sciences, University of Roehampton, London, UK; School of Clinical Science, Faculty of Health Science, Queensland University Technology, Brisbane, Australia; Department of Physiotherapy, Faculty of Health Sciences, University of Malaga, Malaga, Spain

**Keywords:** Pennation angle, Muscle thickness, Sonomyography, Electromyography, Rectus femoris, Vastus lateralis, Isometric contraction

## Abstract

**Objectives:**

The purpose of the study was to establish regression equations that could be used to predict muscle thickness and pennation angle at different intensities from electromyography (EMG) based measures of muscle activation during isometric contractions.

**Design:**

Cross-sectional study.

**Methods:**

Simultaneous ultrasonography and EMG were used to measure pennation angle, muscle thickness and muscle activity of the rectus femoris and vastus lateralis muscles, respectively, during graded isometric knee extension contractions performed on a Cybex dynamometer. Data form fifteen male soccer players were collected in increments of approximately 25% intensity of the maximum voluntary contraction (MVC) ranging from rest to MVC.

**Results:**

There was a significant correlation (P < 0.05) between ultrasound predictors and EMG measures for the muscle thickness of rectus femoris with an R^2^ value of 0.68. There was no significant correlation (P > 0.05) between ultrasound pennation angle for the vastus lateralis predictors for EMG muscle activity with an R^2^ value of 0.40.

**Conclusions:**

The regression equations can be used to characterise muscle thickness more accurately and to determine how it changes with contraction intensity, this provides improved estimates of muscle force when using musculoskeletal models.

## Introduction

The most commonly used approach for the assessment of skeletal muscle is EMG and can provide the biochemical and electrophysiological features of the of the muscle (Brody et al. 1991; Cifrek et al. 2009). The analysis of EMG signals is a valuable approach to study muscular contractions (Merletti and Parker 2004). Many studies have reported the relationship between the surface EMG (sEMG) and muscle force, length and muscle fibre conduction velocity (McGinnis 2005; Merletti and Lo Conte 1997; Lieber 2002).

The ultrasound image has been widely used to assess human muscles also referred to as sonomyography (Shi et al. 2007). This has been used to measure the changes in muscle thickness Zheng et al. 2006; Brancaccio et al. 2008; Legerlotz et al. 2010), muscle fibre pennation angle (Rutherford and Jones 1992; Lieber and Friden 2000; Klimstra et al. 2007), muscle fascicle length (Fukunaga et al. 1997a, and muscle size (Fukunaga et al. 1997b; Reeves et al. 2004) during static and dynamic conditions. The sonographic image of normal skeletal muscle is hypoechoic with linear, parallel, hyperechoic echoes. Muscle is generally less echogenic than subcutaneous fat or tendons.

Since the muscular architectural parameters are a primary determination of muscle function, they could potentially provide a non-invasive and safe method of recording activities from superficial muscles without the complication of the cross-talk from adjacent muscles suffered in the sEMG analysis (Hodges et al. 2003). Several studies have examined the changes in the muscular architectural parameters. One study found differences in pennation angle and thickness in the triceps brachii among bodybuilders and normal subjects (Kawakami et al. 1993). Bleakney and Maffulli (2002) (Bleakney and Maffulli 2002) found differences in muscle thickness and pennation angle by the detraining in the quadriceps muscles compared with the contralateral after a fracture in the limbs. While Rutherford and Jones (1992) found no difference in the pennation angle after a three month of strengthening programme of the quadriceps muscles.

Most recently, some studies have shown the relationship between EMG activity and muscular architecture. One study showed a positive relationship between the change of muscle thickness and sEMG activity of the abdominal (Bleakney and Maffulli 2002), and another study showed a relationship between muscle pennation angle and sEMG during ankle isometric contractions (McMeeken et al. 2004). The combination of muscular architecture and sEMG activity studies provide more information about skeletal muscle. We are not aware of any study that has used ultrasound and sEMG to assess the relationship between ultrasound parameters and sEMG from the quadriceps muscles during different level of isometric contractions.

Therefore, the aim of the study was to assess the relationship between muscle thickness of rectus femoris and pennation angle of vastus lateralis from EMG based measures of muscle activation during isometric contractions of different strength intensities (100%, 75%, 50%, 25%). We hypothesised that pennation angle and muscle thickness would increase with EMG, and the strength of the relationship would relate to the changes in pennation angle and muscle thickness form rest to MVC.

## Methods

### Participants

Fifteen healthy male subjects (semi-professional football players) from various local football teams were recruited for this study. The subjects were male and right leg dominant (age 24.4 ± 3.2 years; height 181.1 ± 6.4 cm; body mass 80.5 ± 11.6 kg; mean ± SD). Subjects suffering from any lower limb injury within the previous three months of the assessment were excluded from the study. No participants had any previous history of neuromuscular disorder and each gave written informed consent prior to the experiment. Ethical approval for the study was gained according to the guidelines of University of Roehampton.

### Experimental procedure

Before the test, each subject carried out a 10-minute warm-up including static stretching of the quadriceps muscles, free standing squats and jogging. Knee extension torque was measured isometrically using the Cybex dynamometer with HUMAC Norm software (Cybex Norm Testing & Rehabilitation System, Cybex Norm Inc., Ronkonkoma, USA). The subject was then seated in the adjustable chair and was secured with safety straps around the waist and across the chest to limit any changes in position during testing. The lateral condyle of the knee joint was visually aligned with the centre of the rotational axis of the dynamometer. The ankle cuff on the dynamometer’s lever arm was placed 2 cm proximal to the lateral malleolus, this allowed subjects to exert maximal forces without receiving any discomfort at the shin. Knee extension contractions were performed at a knee flexion angle of 75° flexion. The right leg was chosen for measurements, which was the dominant leg in all subjects.

The location for electrodes placement was taken from the European Recommendations for Surface Electromyography (SENIAM 1999) and ground electrode was placed in the wrist of subject. The subject was marked up using a washable marker pen and a razor was used to remove any hair on the skin that was cleansed with alcohol wipes and dried. The electrode for the vastus lateralis was placed 2/3 on the line from the anterior spina iliaca superior to the lateral side of the patella. The electrode for the rectus femoris was placed at 1/2 on the line from the anterior spina iliaca superior to the superior part of the patella.

After several familiarization contractions, each subject was asked to extend the knee against the lever actuator of the dynamometer by increasing the contraction force little by little to the maximal contraction and then relaxing gradually. A real time visual reference for the maximum torque was displayed on the isokinetic dynamometer computer screen that assisted with targeting. The maximal torque value was considered as the maximal voluntary contractions (MVC). Two repeated voluntary contractions were performed continuously within each trial with a 5 minutes rest between trials. The assessment included two trials at rest, maximum voluntary contraction (MVC), 75%, 50% and 25% for the rectus femoris and vastus lateralis whilst simultaneously recording sEMG and US images with support of a trigger system. Once the test procedure was completed, a 5-minute cool down was undertaken by each subject. This consisted of slow walking and static stretching of the quadriceps muscles for a duration of 15–20 seconds.

### Data acquisition

The Cybex dynamometer was used to measure the isometric peak torques at 75° of knee flexion. Verbal encouragement and feedback was given to each subject for each contraction. A value of ±5 Nm of the actual torque target was accepted and recorded. Once the subject reached the required torque target point for each increment, they were instructed to hold the contraction for five seconds before being told to relax.

EMG data was measured using Biometrics DataLink Software (Ladysmith, VA, USA). sEMG activity was recorded with the use of a data logger with the start of the recording taking place just before the contraction and stopped when the subject had finished contracting and was in a relaxed position.

The Ultrasound scan was measured using Sonosite M-turbo with a high frequency transducer type HFL38x (13–6 MHz) (Bothell, WA, USA). The same operator performed all measurements. The thickness of the rectus femoris was measured with the probe placed in the transverse plane, and the angles of pennation of the vastus lateralis with the probe placed in the sagittal plane. A transducer was used to record the ultrasound images and placed inferior to the surface electrode placements of the rectus femoris and vastus lateralis. Liberal amounts of conductive gel were applied to the skin and the probe to obtain a clear and good quality image.

An external trigger with sEMG synchronized the ultrasound images once the subject had met the torque target value and was holding the contraction for 5 seconds. Two trials were for each muscle, beginning with RF muscle. Resting periods between each trial for the subject lasted for 5 minutes before the second trial began.

### Data analysis

**Figure 1:**
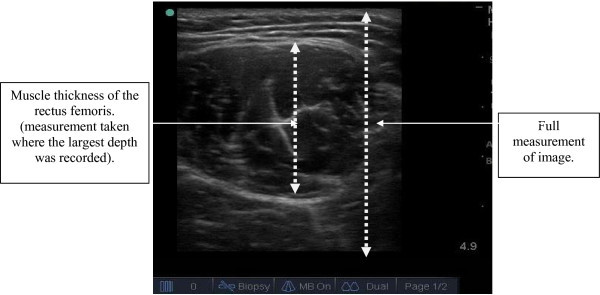
**Image showing the measurement of muscle thickness for the rectus femoris.**

**Figure 2:**
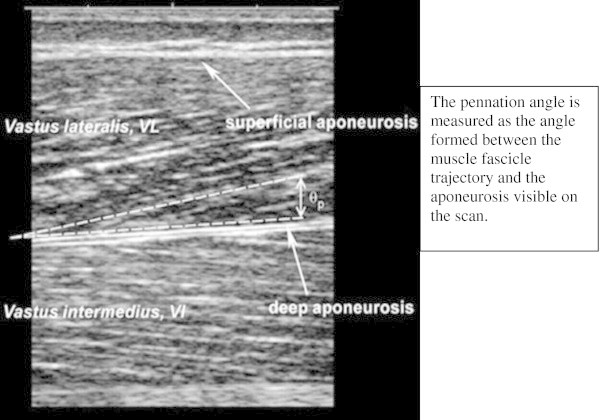
**Image showing the measurement of pennation angle for the vastus lateralis.**

The sEMG data were sampled at 1000 Hz, and the data for the two trials at rest, MVC, 75%, 50% and 25% were averaged for both muscles. The signals were further processed by low pass filtering the signal with a Butterwotrth filter at a cut-off frequency of 500 Hz. The root mean square (RMS) sEMG amplitude was measured for 5 seconds and was then normalized to the average from MVC, normalized sEMG was used as a measure of muscle activation during contraction. Normalized sEMG ranged between 0 and 1.0. Ultrasound images for the two trials at rest, MVC, 75%, 50% and 25% were averaged for both the rectus femoris and vastus lateralis muscles. The thickness was measured from the anterior aspect of rectus femoris to the fascia (Figure 1). The pennation angle was determined from the angle between the fascia and the muscle fibres (Figure 2). All measurements were taken using the ImageJ software (National Institutes of Health, Bethesda, Maryland).

### Statistical analysis

Statistical analysis was performed using SPSS 15.0 statistical package for Windows. Linear regressions were used for each muscle with a p-value less than 0.05 considered statistically significant. EMG data were used alone as input and pennation angle and muscle thickness how output.

## Results

Mean and Standard Deviation (SD) for rectus femoris and vastus lateralis sEMG muscle activity and architecture parameters values for all 15 subjects are shown in Table 1. An increase in muscle force leads to an increase in muscle thickness for the rectus femoris and pennation angle for the vastus lateralis with the largest difference recorded from rest to MVC (100%).

**Table 1:** **Mean±SD data of the sEMG (mv) and muscle thickness (mm) and pennation angle (°) during levels of intensity**

	Rest	25%	50%	75%	100%
**Vastus lateralis**					
**Average ± SD (mv)**	0.003 ± 0.002	0.062 ± 0.028	0.152 ± 0.061	0.266 ± 0.122	0.390 ± 0.151
**Average ± SD (°)**	15.1 ± 3.81	15.7 ± 3.77	17.6 ± 4.06	18.8 ± 4.52	20.7 ± 4.44
**Rectus femoris**					
**Average ± SD (mv)**	0.008 ±0.008	0.052 ± 0.020	0.149 ± 0.0658	0.293 ± 0.124	0.431 ± 0.185
**Average ± SD (mm)**	28.8 ± 3.28	32.2 ± 3.97	33.3 ± 4.21	33.4 ± 4.04	33.9 ± 4.3

**Figure 3:**
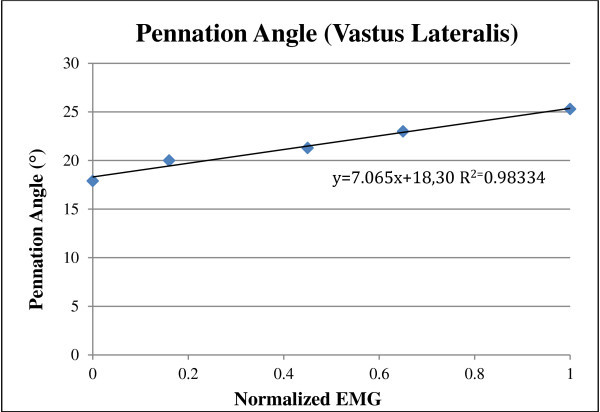
**Best measures for Pennation Angle and EMG for one subject.**

**Figure 4:**
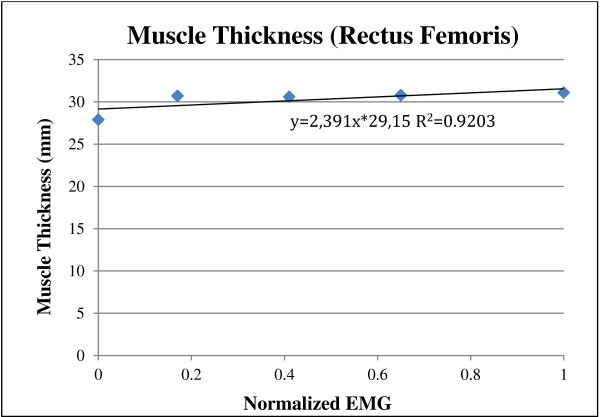
**Best measures for Muscle Thickness and EMG for one subject.**

Examples of the best measures for ultrasound pennation angle and muscle thickness with sEMG muscle activity for one subject are shown in Figures 3 and 4. It is important to note that the slope and fit of the line varies from normalized data of one subject to another because of inter-subject variability. For this reason, pennation angle and muscle thickness at rest and MVC were included in the anlaysis so that subject specific values can be predicted for subjects with similar normalised EMG.

**Figure 5:**
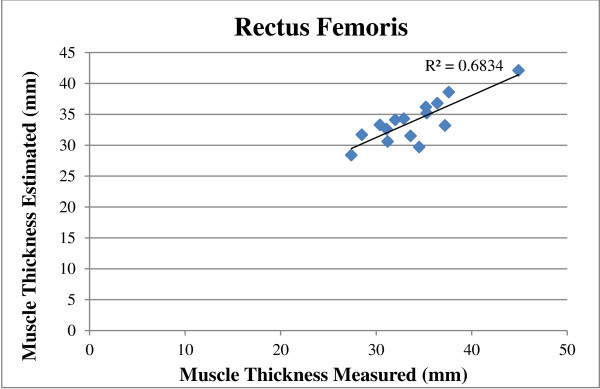
**Muscle thickness estimated and measured at 100% MVC for the rectus femoris for the whole group.**

**Figure 6:**
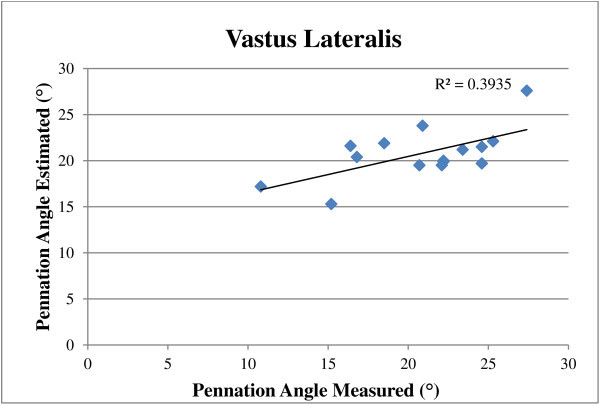
**Pennation angle estimated and measured at 100% MVC for the vastus lateralis for the whole group.**

The regression equations for EMG and ultrasound fitted significant (p < 0.05) with R^2^ value of 0.68 for ultrasound muscle thickness of the rectus femoris, (Figure 5). However, for pennation angle of the vastus lateralis, the regression equations for EMG and ultrasound was not significant (p > 0.05) with R^2^ values of 0.40, (Figure 6).

## Discussion

This study showed that there was a significant correlation between EMG muscle activity and muscle thickness for the rectus femoris muscle (p < 0.05). In addition, there was no significant correlation between EMG muscle activity and pennation angle for the vastus lateralis muscle (p > 0.05).

Manal et al. (2008) found a significant correlation between EMG and pennation angles of the ankle plantar and dorsiflexors including the tibialis anterior, lateral gastrocnemius, medial gastrocnemius, and soleus muscles with R^2^ values of 0.76, 0.82, 0.82 and 0.87 which shows a strong relationship between the two parameters of EMG and pennation angle. Hodges et al. (2003) used ultrasound as a method to estimate muscle activity in several muscles including tibialis anterior, biceps brachii, brachialis, transverses abdominis, obliquus internus abdominis and obliquus externus abdominis during isometric contractions. Their study found a relationship in the tibialis anterior muscle with a significantly higher correlation between EMG and pennation angle with an R^2^ value of 0.96 and between EMG and muscle thickness was also significant with an R^2^ value of 0.75 (Hodges et al. 2003). However, methodological differences can be related to the differences between by Manal et al. (2008) and Hodges et al. (2003) findings. As mentioned previously, the joint angle is an important factor in conducting strength assessments. Both studies used the tibialis anterior muscle of the ankle joint for assessing muscular strength at different intensities. The study by Manal et al. (2008) collected data with the ankle in 30° of plantar flexion which was stated as placing the muscle near the optimal fiber length whereas Hodges et al. (2003) collected data with the ankle in a neutral position. Hodges et al. (2003) used finer increments of % MVC, whereas in comparison to the current study and research conducted by Manal et al. (2008) percentage increments of 25% from rest to MVC were used (25%, 50%, 75%). The Hodges study shows that a range of architectural parameters of human muscles is nonlinearly related to muscle activity, but they doesn’t study the quadriceps muscle (Hodges et al 2003). In contrast, a linear relationship between muscle activity and changes in muscle geometry has been reported for certain muscles (e.g., medial gastrocnemius) (Narici et al 1996).

The findings of the current study suggest that as muscle contraction level increases, muscle thickness and pennation angle also increase. This is supported by previous literature from Rudroff et al. (2008) who found increases in brachialis thickness (27.7 to 30.9 mm) and pennation angle (10.9 to 16.5°) of the elbow flexors. Their testing procedure involved fatiguing contractions at 20% of maximal voluntary contraction force, however, this was not associated with the increase in intramuscular EMG amplitude. In comparison to the current study, EMG muscle activity recorded a significant increase with muscle contraction intensity from rest, 100%, 75%, 50%, 25% with values of 0.0033, 0.3901, 0.2661, 0.1526, 0.0621 (mv) respectively. Furthermore, it shows that there is a relationship between EMG and ultrasound in relation to increase EMG muscle activity and muscle thickness and pennation angles at different intensities.

Ghori et al. (1995) investigated the relationship between torque and EMG activity of the vastus lateralis during isokinetic concentric and eccentric contractions at maximum effort with a fixed velocity of 30°s^-1^ through 80°- 20° of knee flexion. They found no significant differences in EMG activity between the left and right vastus lateralis or between eccentric and concentric contractions at any joint angle range. The possible reasons for this can relate to electrode placement, properties of underlying tissue such as fat or fiber type characteristics of the muscle (Ghori et al. 1995). Electrode placement is an important factor for recording muscle activity. In the current study, references for the location of electrode placement were taken from the European Recommendations for Surface Electromyography as well as using a reference for the ultrasound probe to provide accurate electrode and probe placements.

Brancaccio et al. (2008) found significant increases in pennation angles of the vastus lateralis which were significantly greater after exercising to exhaustion in all subjects with an average of 14.4° compared to 12.8° when the subjects were at rest. The current study recorded an increase in pennation angle for the vastus lateralis with a mean value of 15.1° for the subjects at rest and 20.7° for the mean value at MVC.

Buchanan et al. (2004) stated that contractile effort was reflected in the magnitude of the electromyogram, therefore the EMG signal might be a predictor of muscle thickness and pennation angle change. The findings of the current study revealed that the relationship between EMG and muscle thickness was fairly linear with a significant correlation for the rectus femoris (p < 0.05). The rectus femoris recorded the strongest relationship with an R^2^ value of 0.68 using EMG regression equations to predict muscle thickness. There was no significant correlation between EMG muscle activity predictors for ultrasound pennation angle with an R^2^ value of 0.40 (p > 0.05).

Further limitations of the study can relate to only one joint angle was recorded, that pennation angles were measured only from one muscle and the recording of EMG for the vastus lateralis muscle as it is likely that all components of the quadriceps musculature acted together during the contractions. Image scans were taken from one particular instant of effort while EMG measures were averaged across 5 seconds. In addition, the relationships were fitted for each muscle during separate contractions. For the generalizability of the results expanded the sample females and lesser-trianed individuals must be included. The use of the isokinetic dynamometer to provide isometric torque values was also a limitation of the study. Once the subject was holding the contraction for five seconds after reaching the required torque value, it was difficult to identify if the subject was still holding the contraction at the required amount of effort. Only a visual reference was undertaken to make sure the subject was still contracting the muscles, as this would have had an effect on the ultrasound image as well as the EMG signal.

## Conclusion

The relationship between EMG muscle activity and muscle thickness demonstrated a strong correlation. These findings suggest that EMG based measures can be used to predict muscle thickness and ultrasound based measures can be used to predict EMG muscle activity. This is interesting because the two assessment tools of EMG and ultrasound measure different aspects of muscle function. The study also found no significant correlation between EMG muscle activity and ultrasound pennation angle for the vastus lateralis.

The results of this study establish the regression equations to predict muscle thickness of the rectus femoris from EMG. Incorporating this relationship into a model may better represent the architucture of this muscle which dominates knee extension. The equations are easy to implement and can improve musculoskeletal modeling force estimates for the primamary knee extensors.

### Practical implications

-  Regression equations could be used to predict muscle architecture from EMG-based measures.
-  The rectus femoris recorded the strongest relationship between EMG muscle activity and muscle thickness.
-  The above relationship could, therefore, be used to develop musculoskeletal modelling force estimates for the quadricep muscles.
-  No financial support was received for this study.
